# Potential formula of an *m* × *n* globe network and its application

**DOI:** 10.1038/s41598-018-27402-4

**Published:** 2018-07-02

**Authors:** Zhen Tan, Zhi-Zhong Tan

**Affiliations:** 1 School of Electronics and Information, Nantong University, Nantong, 226019 China; 2 Department of physics, Nantong University, Nantong, 226019 China

## Abstract

Searching for the explicit solutions of the potential function in an arbitrary resistor network is important but difficult in physics. We investigate the problem of potential formula in an arbitrary *m* × *n* globe network of resistors, which has not been resolved before (the previous study only calculated the resistance). In this paper, an exact potential formula of an arbitrary *m* × *n* globe network is discovered by means of the Recursion-Transform method with current parameters (*RT-I*). The key process of *RT* method is to set up matrix equation and to transform two-dimensional matrix equation into one-dimensional matrix equation. In order to facilitate practical application, we deduced a series of interesting results of potential by means of the general formula, and the effective resistance between two nodes in the *m* × *n* globe network is derived naturally by making use of potential formula.

## Introduction

The development of natural science raises many complex new problems and requires people to find the basic method to resolve them. It was found that many problems could be resolved by building a resistor network model. It was first in 1845 (170 years ago), the German scientist Kirchhoff set up the node current law and the circuit voltage law^1^, from then on the basic theory of research resistor network is established. Nowadays, it is found that the resistor network is not only a model but also exists in the real nature. As is well known, Andre Geim and Konstantin Novoselov found the graphene network which is a real-plane resistor network existed in nature, who therefore won the 2010 Nobel Prize in Physics for their investigations. Studies have shown that resistor network model is an interdisciplinary problem, which cannot only be considered as a science, but also a technique, therefore make significance for theories and applications^2–8^.

In the past decades, the research of resistor network has yielded many results, such as the research on classical transport in disordered media^5^, lattice Greens functions^6^, electromigration phenomena in metallic lines^7^, graph theory^8^, resistance distance^9^, infinite network^10,11^, finite network^12^, impedance network^13^, and corner-to-corner resistance^14,15^ and so on. In addition, the calculation of node potential is also important since it has to do with many important problem, such as the transmission line, electrostatic field, IC design, and Laplace equation. In particular, Poisson equation and Laplace equation are the important equations of potential function^16,17^, and the potential theory is about the general theory of solutions to Laplace equation. The potential function has been applied to many interdisciplinary fields which includes the electrical and non-electric fields, such as the fields of electromagnetism, fluid dynamics, and astronomy and so on.

For researching resistor networks, several effective methods for calculating the resistance of resistor networks have been found, such as Cserti^10^ set up the Green function technique to evaluate the resistance of infinite lattices; Wu^12^ formulated a Laplacian matrix method and derived the resistance in arbitrary finite and infinite lattices by means of the eigenvalues and eigenvectors. After some improvements, the Laplacian approach has also been applied to the impedance network^13^ and to the resistor network with zero resistor boundary^18–20^, and to the other resistor networks^21,22^.

In recent years, Tan^23–25^ proposed the Recursion-Transform (RT) method which, depends on the one matrix along one directions, avoids the trouble of the Laplacian method depends on two matrices along two directions. Researchers have resolved a lot of complex resistor network by the RT technique^26–37^, and the results expressed by fractional-order are in the form of a single summation. As a summary, the RT method is expressed by two forms, one form is expressed by current parameters^23–25^, which is simply called the *RT-I* method; another form is expressed by potential parameters^36,37^, which is simply called the *RT-V* method. Very recently, the researchers have also made new progress in the multi-functional N-order resistance network^38,39^, and the result derived by the RT method has also been applied to the impedance network^40^.

**Figure 1:**
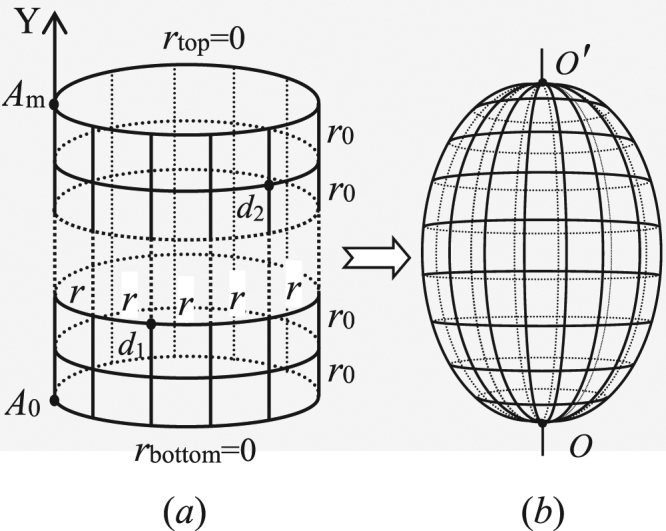
(**b**) is an *m* × *n* globe network which, is topology of a cylindrical network from (**a**), has *n* longitude and *m* − 1 latitude. Bonds in the longitude and latitude directions represent, respectively, resistors *r*_0_ and *r*.

According to research information obtained from the above literature, we know the computation of equivalent resistance has made great progress^2–40^, but the calculation of the nodal potential has been a difficult problem and has not been resolved until a recent research of literature^36,37^. Ref.^36^ gives the precise potential formulae of the fan and cobweb networks by the *RT-V* method for the first time, next ref.^37^ studies the potential formula of nonregular fan network by the *RT-V* method. However, due to the diversity of network types, there are still many potential of the resistor networks needs to be calculated. Ref.^27^ had ever calculated the effective resistance of the globe network as shown in Fig. 1, but the electric potential of the globe network hasn’t been resolved before. In this paper we are going to study the potential equation of the arbitrary globe network by the *RT-I* method, which is different from the *RT-V* method used in refs^36,37^.

Reviewing the main calculation process of *RT* method, when you research the globe network by means of the *RT-I* method, referring Fig. 1 that it inevitably produces a tridiagonal matrix^25,27^

1 $${{\bf{B}}}_{m}=[\begin{array}{ccccc}{\rm{2}}+b & -b & 0 & 0 & 0\\ -b & 2(1+b) & -b & 0 & 0\\  & \vdots \,\,\,\,\, & \ddots  & \,\,\vdots  & \\ 0 & 0 & -b & 2(1+b) & -b\\ 0 & 0 & 0 & -b & {\rm{2}}+b\end{array}].$$

where b = *r*/*r*_0_. Using the *RT-I* method to resolve matrix equations built for resolving the branch current, we need to transform **B**_*m*_ by **P**_m_**B**_m_ = **T**_*m*_**P**_*m*_, where we first must work out the eigenvalues of matrix **B**_*m*_, taking

2 $${\rm{\det }}|{{\bf{B}}}_{m}-t{{\bf{E}}}_{m}|=0$$

we get

3 $${t}_{i}=2+2b-2b\,\cos \,{\theta }_{i},$$

where *θ*_*i*_ = (*i* − 1)*π*/*m*. Thus one can obtain the eigenvectors of matrix by following identity

4 $${{\bf{P}}}_{m}{{\bf{B}}}_{m}={\rm{diag}}({t}_{1},{t}_{2},\cdots ,{t}_{m}){{\bf{P}}}_{m}.$$

We therefore obtain the eigenvectors

5 $${{\bf{P}}}_{m}=(\begin{array}{cc}\begin{array}{cc}1/\sqrt{2} & 1/\sqrt{2}\\ \cos ({v}_{1}{\theta }_{2}) & \cos ({v}_{2}{\theta }_{2})\end{array} & \begin{array}{cc}\cdots  & 1/\sqrt{2}\\ \cdots  & \cos ({v}_{m}{\theta }_{2})\end{array}\\ \begin{array}{cc}\vdots  & \vdots \\ \cos ({v}_{1}{\theta }_{m}) & \cos ({v}_{2}{\theta }_{m})\end{array} & \begin{array}{cc}\ddots  & \vdots \\ \cdots  & \cos ({v}_{m}{\theta }_{m})\end{array}\end{array}),$$

where *v*_*k*_ = *k* − 1/2. Meanwhile, we obtain the inverse matrix after a simple calculation,

6 $${{\bf{P}}}_{m}^{-1}=\frac{2}{m}[\begin{array}{cccc}1/\sqrt{2} & \cos ({v}_{1}{\theta }_{2}) & \cdots  & \cos ({v}_{1}{\theta }_{m})\\ 1/\sqrt{2} & \cos ({v}_{2}{\theta }_{2}) & \cdots  & \cos ({v}_{2}{\theta }_{2})\\ \vdots  & \vdots  & \ddots  & \vdots \\ 1/\sqrt{2} & \cos ({v}_{m}{\theta }_{m}) & \cdots  & \cos ({v}_{m}{\theta }_{m})\end{array}],$$

Equations (1–6) will be the preparatory knowledge to study the globe network by means of the *RT-I* method in the following.

The organization of this paper is as follows: In Sec. 1, we describe the RT method and explain the reasons for research. In Sec. 2, we present potential formula of the globe network. In Sec. 3, we gave the derivation by the RT-I method. In Sec. 4, deducing many specific results of potential. Sec. 5 gives a summary and discussion of the method and results.

## Results

**Figure 2:**
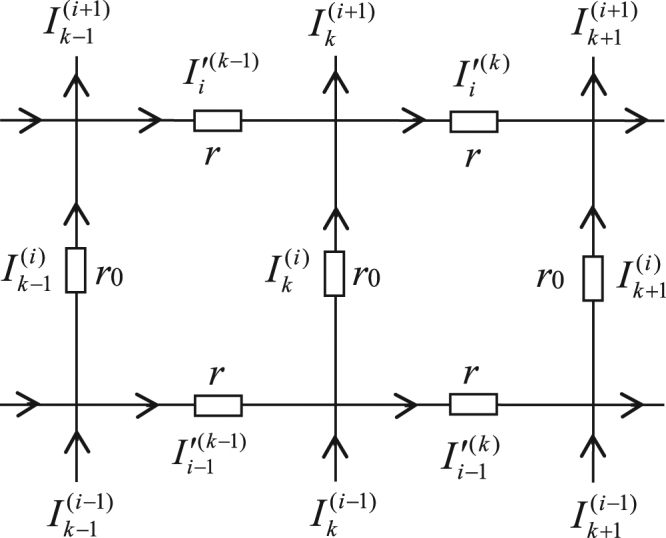
Segment of the globe network with current directions and parameters.

Consider an general *m* × *n* globe network with resistors *r* and *r*_0_ in the respective latitude and longitude directions, where *n* and *m* are the numbers of resistors along latitude and longitude directions as shown in Fig. 1. Selecting the point *O* as the origin of the coordinate system, and a longitude act as Y axis. Assuming nodes coordinate of the network is {*x*, *y*} and potential of node d(*x*, *y*) is ${U}_{m\times n}(x,y)={V}_{x}^{(y)}$ as shown in Fig. 2 (Fig. 2 is a sub-network of the globe network).

Assuming the current *J* goes from *d*_1_(*x*_1_, *y*_1_) to *d*_2_(*x*_2,_ *y*_2_), and *U*_*m*×*n*_(0, 0) = 0, the potential of any node in the *m*×*n* globe network is

7 $$\frac{{U}_{m\times n}(x,y)}{J}=\frac{y({y}_{1}-{y}_{2})}{mn}{r}_{0}+\frac{2r}{m}\sum _{i=2}^{m}\frac{{\beta }_{{x}_{1},x}^{(i)}{S}_{{y}_{1},i}-{\beta }_{{x}_{2},x}^{(i)}{S}_{{y}_{2},i}}{{\lambda }_{i}^{n}+{\bar{\lambda }}_{i}^{n}-2}{S}_{y,i},$$

where we define

8 $${S}_{k,i}=\,\sin ({y}_{k}{\theta }_{i}),\,{\theta }_{i}=(i-1)\pi /m,$$

9 $${\beta }_{{x}_{s},{x}_{k}}^{(i)}={F}_{n-|{x}_{s}-{x}_{k}|}+{F}_{|{x}_{s}-{x}_{k}|},\,{F}_{k}^{(i)}=({\lambda }_{i}^{k}-{\bar{\lambda }}_{i}^{k})/({\lambda }_{i}-{\bar{\lambda }}_{i}),$$

10 $$\begin{array}{c}{\lambda }_{i}=1+b-b\,\cos \,{\theta }_{i}+\sqrt{{(1+b-b\cos {\theta }_{i})}^{2}-1},\\ {\bar{\lambda }}_{i}=1+b-b\,\cos \,{\theta }_{i}-\sqrt{{(1+b-b\cos {\theta }_{i})}^{2}-1}\end{array}$$

with *b* = *r*/*r*_0_.

In particular, when *n* → ∞ but *m* is finite, Fig. 1 is called a semi-infinite network, the potential formula of the node *d*(*x*, *y*) in the *m* × ∞ globe network can be written as

11 $$\frac{{U}_{m\times \infty }(x,y)}{J}=\frac{r}{m}\sum _{i=2}^{m}\frac{{\bar{\lambda }}_{i}^{|{x}_{1}-x|}{S}_{1,i}-{\bar{\lambda }}_{i}^{|{x}_{2}-x|}{S}_{2,i}}{\sqrt{{(1-b-b\cos {\theta }_{i})}^{2}-1}}{S}_{y,i}.$$

Formulae (7) and (11) are potential theorem of any point *d*(*x*, *y*) in a general *m* × ∞ globe network, which are discovered for the first time.

## Method

### A general idea and design

To compute the node potential of a general node in the *m* × *n* globe network, we inject a current *J* into the network at *d*_1_(*x*_1_, *y*_1_) and exit the current at *d*_2_(*x*_2_, *y*_2_). Denoting the branch currents in all segments of the network as shown in Fig. 2. All longitude currents are: ${I}_{k}^{(1)}$, ${I}_{k}^{(2)}$, … ${I}_{k}^{(m)}$ (0 ≤ *k* ≤ *n*). We assume *U*(0, 0) = 0, then by Ohm’s law the node potentials relative to the pole *O*(0, 0) can be written as

12 $${U}_{m\times n}(x,y)=U(0,0)-{r}_{0}\sum _{i=1}^{y}{I}_{x}^{(i)}=-\,{r}_{0}\sum _{i=1}^{y}{I}_{x}^{(i)},$$

where ${I}_{x}^{(i)}$ denotes currents along the longitude direction via *d*(*x*, *y*). Why does Eq. (6) take “−” is that we set the current direction as shown in Fig. 2, as you know the potential is going down along the direction of the current. How to solve the branch current parameters ${I}_{k}^{(i)}$ is the key to the problems. We are going to resolve the problem by using the *RT-I* approach^23–25^.

### Modeling Recurrence Relations

We make use of a sub-network of Fig. 2 and Kirchhoff’s law to analyze the resistor network, and to set up the nodes current equations and the meshes voltage equations. Consider the two rectangular meshes and six nodes as shown in Fig. 2. When injecting the electric current *J* at node of *d*_1_(*x*_1_, *y*_1_) and exiting *J* at node of *d*_2_(*x*_2_, *y*_2_), we obtain the following equations of only containing current parameters along longitude direction by eliminating the current parameters along latitude direction.

13 $$\begin{array}{c}\,\,\,{I}_{k+1}^{(1)}=(2+b){I}_{k}^{(1)}-b{I}_{k}^{(2)}-{I}_{k-1}^{(1)}-b{I}_{1}{\delta }_{k,x},\,i=1\\ {I}_{k+1}^{(i)}=(2+2b){I}_{k}^{(i)}-b{I}_{k}^{(i-1)}-b{I}_{k}^{(i+1)}-{I}_{k-1}^{(i)}-b{I}_{i}{\delta }_{k,x},\,1 < i < m\\ \,\,\,{I}_{k+1}^{(m)}=(2+b){I}_{k}^{(m)}-b{I}_{k}^{(m-1)}-{I}_{k-1}^{(m)}-b{I}_{m}{\delta }_{k,x},\,i=m\end{array}$$

where *b* = *r*/*r*_0_, and *I*_*i*_ is the external current source, and

14 $${I}_{i}=-\,J({\delta }_{i,{y}_{1}}-{\delta }_{i,{y}_{1}+1})+J({\delta }_{i,{y}_{2}}-{\delta }_{i,{y}_{2}+1})$$

and the delta function *δ*_*i*,*y*_ means: *δ*_*i*,*y*_(*y* = *i*) = 1, *δ*_*i*,*y*_(*y* ≠ *i*) = 0.

By Eqs (13) and (14), we obtain a general matrix equation

15 $${{\bf{I}}}_{k+1}={{\bf{B}}}_{m}{{\bf{I}}}_{k}-{{\bf{I}}}_{k-1}-b{{\bf{H}}}_{y}{\delta }_{k,x},$$

where ***I***_*k*_ and ***H***_*x*_ are the *m* × 1 column matrixes, and can be written as

16 $${{\bf{I}}}_{k}={[{I}_{k}^{(1)},{I}_{k}^{(2)},{I}_{k}^{(3)},\cdots ,{I}_{k}^{(m)}]}^{T},$$

17 $${({H}_{1})}_{i}=J(-{\delta }_{i,{y}_{1}}+{\delta }_{i,{y}_{1}+1}),\,{({H}_{2})}_{i}=J({\delta }_{i,{y}_{2}}-{\delta }_{i,{y}_{2}+1})$$

where matrix **B**_*m*_ in Eq. (15) is given by Eq. (1).

Next we consider the constraint conditions related the left and right edges. Based on the two sides of the Y axis to set up the constraint equation by Kirchhoff’s laws, we get

18 $${{\bf{I}}}_{n-1}+{{\bf{I}}}_{1}={{\bf{B}}}_{m}{{\bf{I}}}_{0},$$

19 $${{\bf{I}}}_{0}={{\bf{I}}}_{n}={{\bf{B}}}_{m}{{\bf{I}}}_{n-1}-{{\bf{I}}}_{n-2}.$$

where matrix **B**_*m*_ is given by (1), and **I**_0_ = **I**_*n*_ is because of cycle.

Above Eqs (15–19) are all equations we need to calculate the branch current and the node potential of the *m* × *n* globe network, but we cannot directly resolve the above equations since they have many variables. Thanks to the RT technique which provides us an indirect method to resolve the equations.

### Matrix Transform Approach

We first consider to solve the matrix Eq. (15). To realize the idea, we’re assuming an undetermined square matrix ***P***_*m*×*m*_, and multiplying (14) from the left side by matrix ***P***_*m*_,

20 $${{\bf{P}}}_{m}{{\bf{I}}}_{k+1}={{\bf{P}}}_{m}{{\bf{B}}}_{m}{{\bf{I}}}_{k}-{{\bf{P}}}_{m}{{\bf{I}}}_{k-1}-b{{\bf{P}}}_{m}{{\bf{H}}}_{x}{\delta }_{k,x}.$$

By Eqs (3) and (5), we have the eigenvalues ${t}_{i}=2+2b-2b\,\cos \,{\theta }_{i}$, and have eigenvectors (5), we therefore can transform Eq. (20), such as, by Eqs (4) and (20) we appoint

21 $${{\bf{P}}}_{m}{{\bf{I}}}_{k}={{\bf{Y}}}_{k}\,{\rm{and}}\,{{\bf{I}}}_{k}={{\bf{P}}}_{m}^{-1}{{\bf{Y}}}_{k}.$$

where the *m* × 1 column matrix **Y**_*m*_ is

22 $${{\bf{Y}}}_{k}={[{Y}_{k}^{(1)},{Y}_{k}^{(2)},{Y}_{k}^{(3)},\cdots ,{Y}_{k}^{(m)}]}^{{\rm{T}}}.$$

Thus, applying Eqs (4) and (21) to Eq. (20), yields

23 $${Y}_{k+1}^{(i)}={t}_{i}{Y}_{k}^{(i)}-{Y}_{k-1}^{(i)}-Jb{\zeta }_{y,i}{\delta }_{k,x},$$

where

24 $$\begin{array}{ccc}{\zeta }_{1,i} & = & -\,2\,\sin (\frac{1}{2}{\theta }_{i})\,\sin ({y}_{1}{\theta }_{i}),\\ {\zeta }_{2,i} & = & 2\,\sin (\frac{1}{2}{\theta }_{i})\,\sin ({y}_{2}{\theta }_{i}).\end{array}$$

Next, we imitate Eq. (23) to transform Eqs (18) and (19) by making use of **P**_*m*_, we obtain

25 $${Y}_{n-1}^{(i)}+{Y}_{1}^{(i)}={t}_{i}{Y}_{0}^{(i)}.$$

26 $${Y}_{0}^{(i)}={Y}_{n}^{(i)}={t}_{i}{Y}_{n-1}^{(i)}-{Y}_{n-2}^{(i)}.$$

At this point, we obtained the simple linear equations of (23), (25) and (26) which can be derived easy. We will give the general solution and special solution of the difference Eq. (23) in the following.

### General solutions of matrix equations

In this section, we are going to derive the exact solution of ${Y}_{k}^{(i)}$ by Eqs (23), (25) and (26). Assuming ${\lambda }_{i},\,{\bar{\lambda }}_{i}$ are the characteristic roots of the Eq. (23) for *Y*_*k*_, then we obtain Eq. (10). Due to the particularity of the eigenvalues in Eq. (3), we have to consider two cases of *i* = 1 and *i* ≥ 2.

When *i* = 1, there be *θ*_1_ = 0, from Eq. (3) we have the eigenvalue *t*_1_ = 2, so we need to consider the additional solution of Eq. (23) under this condition. From Eq. (23) and Eqs (25, 26), we obtain

27 $${Y}_{0}^{(1)}={Y}_{1}^{(1)}=\cdots ={Y}_{n-1}^{(1)}.$$

Using Eq. (5) and Eq. (21), we have

28 $$\sum _{i=0}^{n-1}{Y}_{i}^{(1)}=\frac{1}{\sqrt{2}}\sum _{k=1}^{m}\sum _{i=0}^{n-1}{I}_{i}^{(k)},$$

Based on the input and output position of current in the circuit (the current *J* flows from a node at latitude *y*_1_ to a node at latitude *y*_2_), we obtain the piecewise summation

29 $$\sum _{i=0}^{n-1}{I}_{i}^{(k)}=\{\begin{array}{cc}J, & {y}_{1} < i\le {y}_{2}\\ 0, & {\rm{otherwise}}\end{array},$$

Substituting Eq. (27) and (29) into (28) yields

30 $${Y}_{k}^{(1)}=\frac{J({y}_{2}-{y}_{1})}{n\sqrt{2}}.(0\le k < n)$$

When *i* ≥ 2, solving Eqs (23–26), we achieve the universal solution of the ${Y}_{k}^{(i)}$ with current parameters (*i* ≥ 2)

31 $${Y}_{x}^{(i)}=bJ\frac{{\beta }_{{x}_{1},x}^{(i)}{\zeta }_{1,i}+{\beta }_{{x}_{2},x}^{(i)}{\zeta }_{2,i}}{{\lambda }_{i}^{n}+{\bar{\lambda }}_{i}^{n}-2},\,(0\le x\le n)$$

where ${\beta }_{{x}_{s},x}^{(i)}={F}_{n-|{x}_{s}-x|}+{F}_{|{x}_{s}-x|}$ and ${F}_{k}^{(i)}=({\lambda }_{i}^{k}-{\bar{\lambda }}_{i}^{k})/({\lambda }_{i}-{\bar{\lambda }}_{i})$, and *ζ*_1,*i*_, *ζ*_2,*i*_ are given by Eq. (24).

Thus the key parameters ${X}_{k}^{(i)}$ has been uniformly expressed by Eqs (30) and (31). Equation (30) together with Eq. (31) are all general solutions of matrix equations. Based on the solutions of ${X}_{k}^{(i)}$ that we can derive the branch current and the node potential by means of Eq. (21).

### Derivation of potential function

In this section, we first derive the solutions of branch currents by the above results, then derive the potential distribution. From Eq. (21) we are easy to obtain ${I}_{k}^{(i)}$ by making use of the inverse transformation of ${{\bf{I}}}_{k}={{\bf{P}}}_{m}^{-1}{{\bf{Y}}}_{k}$. By Eqs (6) and (21) we obtain

32 $${I}_{x}^{(j)}=\frac{2}{m}(\frac{1}{\sqrt{2}}{Y}_{x}^{(1)}+\sum _{i=2}^{m}{Y}_{x}^{(i)}\cos (j-\frac{1}{2}){\theta }_{i}).$$

Formula (32) is a key current function, in the following all the calculations depends on this formula. Further, we obtain its summations $\sum _{i=1}^{y}{I}_{s}^{(i)}$ needed in Eq. (12) such as summing (32) over *j* from *j* = 1 to *y* yields

33 $$\sum _{j=1}^{y}{I}_{x}^{(j)}=\frac{2}{m}(\frac{y}{\sqrt{2}}{Y}_{x}^{(1)}+\sum _{i=2}^{m}{Y}_{x}^{(i)}\frac{\sin \,y{\theta }_{i}}{2\,\sin ({\theta }_{i}/2)}).$$

where ${Y}_{x}^{(i)}$ is given by Eqs (30) and (31), and the following equation is used,

34 $$2\sum _{j=1}^{y}\cos (j-\frac{1}{2}){\theta }_{i}=\frac{\sin \,y{\theta }_{i}}{\sin ({\theta }_{i}/2)}.$$

Substituting Eqs (30) and (31) into (33), we get

35 $$\sum _{i=1}^{y}{I}_{x}^{(i)}=\frac{Jy({y}_{2}-{y}_{1})}{mn}+\frac{bJ}{m}\sum _{i=2}^{m}(\frac{{\beta }_{{x}_{1},x}^{(i)}{\zeta }_{1,i}+{\beta }_{{x}_{2},x}^{(i)}{\zeta }_{2,i}}{{\lambda }_{i}^{n}+{\bar{\lambda }}_{i}^{n}-2})\frac{\sin (y{\theta }_{i})}{\sin ({\theta }_{i}/2)}.$$

where ${\beta }_{{x}_{s},x}^{(i)}={F}_{n-|{x}_{s}-x|}+{F}_{|{x}_{s}-x|}$ is defined in Eq. (9).

Next, we derive the potential function based on the above results. Because formula (35) is a general solution which is suitable to any conditions, thus substituting (35) into (12), we obtain

36 $$\frac{{U}_{m\times n}(x,y)}{J}=\frac{y({y}_{1}-{y}_{2})}{mn}{r}_{0}-\frac{r}{m}\sum _{i=2}^{m}\frac{\sin (y{\theta }_{i})}{\sin ({\theta }_{i}/2)}(\frac{{\beta }_{{x}_{1},x}^{(i)}{\zeta }_{1,i}+{\beta }_{{x}_{2},x}^{(i)}{\zeta }_{2,i}}{{\lambda }_{i}^{n}+{\bar{\lambda }}_{i}^{n}-2}).$$

Substituting (24) into (36), we therefore obtain formula (7).

In addition, we think about the condition of *n* → ∞ with *m* finite. By Eqs (9 and 10), we get

37 $$\mathop{\mathrm{lim}}\limits_{n\to \infty }\frac{{\beta }_{k,x}^{(i)}}{{\lambda }_{i}^{n}+{\bar{\lambda }}_{i}^{n}-2}=\mathop{\mathrm{lim}}\limits_{n\to \infty }\frac{{F}_{n-|k-x|}+{F}_{|k-x|}^{(i)}}{{\lambda }_{i}^{n}+{\bar{\lambda }}_{i}^{n}-2}=\frac{{\bar{\lambda }}_{i}^{|k-x|}}{{\lambda }_{i}-{\bar{\lambda }}_{i}},$$

Substituting (37) into (7), we obtain

38 $${U}_{m\times n}(x,y)=\frac{2rJ}{m}\sum _{i=2}^{m}(\frac{{\bar{\lambda }}_{i}^{|{x}_{1}-x|}{S}_{{y}_{1},i}-{\bar{\lambda }}_{i}^{|{x}_{2}-x|}{S}_{{y}_{2},i}}{{\lambda }_{i}-{\bar{\lambda }}_{i}}){S}_{y,i}$$

Associating with ${\lambda }_{i}-{\bar{\lambda }}_{i}=2\sqrt{{(1+b-b\cos {\theta }_{i})}^{2}-1}$, substituting to Eq. (38) we therefore obtain Eq. (11).

So far, two general potential formulae (7) and (11) of an *m* × *n* globe network has been proved by the *RT-I* method.

## Discussion

### Applications of the potential formula

Owing to formula (7) is a general potential result of an *m* × *n* globe network, that is to say, the parameters in formula (7) are generalized and contain everything, we are bound to get a series of special and interesting results when taking some special conditions in formula (7). In the following applications we always assume the potential reference at point *O*(0, 0) is *U*(0, 0) = 0.

***Application*** **1**. Consider an arbitrary *m* × *n* globe network as shown in Fig. 1. Assume the electric current *J* outflow the network from the pole *O*(0, 0), and the current *J* input at the node *d*_1_(*x*_1_, *y*_1_), from (7), the potential of an arbitrary node *d*(*x*, *y*) can be write as

39 $$\frac{{U}_{m\times n}(x,y)}{J}=\frac{y{y}_{1}}{mn}{r}_{0}+\frac{2r}{m}\sum _{i=2}^{m}(\frac{\sin ({y}_{1}{\theta }_{i})\,\sin (y{\theta }_{i})}{{\lambda }_{i}^{n}+{\bar{\lambda }}_{i}^{n}-2}){\beta }_{{x}_{1},x}^{(i)},$$

where ${\beta }_{{x}_{1},x}^{(i)}={F}_{n-|{x}_{1}-x|}+{F}_{|{x}_{1}-x|}$ is defined in Eq. (9).

***Application*** **2**. Consider an arbitrary *m* × *n* globe network of Fig. 1. When we inject current *J* at node *d*_1_(*x*_1_, *y*_1_) and exit the current *J* at node *d*_2_(*x*_1_, *y*_2_)(*x*_2_ = *x*_1_), the potential of an arbitrary node *d*(*x*,*y*) is

40 $$\frac{{U}_{m\times n}(x,y)}{J}=\frac{y({y}_{1}-{y}_{2})}{mn}{r}_{0}+\frac{2r}{m}\sum _{i=2}^{m}(\frac{({S}_{{y}_{1},i}-{S}_{{y}_{2},i}){S}_{y,i}}{{\lambda }_{i}^{n}+{\bar{\lambda }}_{i}^{n}-2}){\beta }_{{x}_{1},x}^{(i)},$$

where *S*_*k*,*i*_ is defined in Eq. (8), and ${\beta }_{{x}_{1},x}^{(i)}$ is defined in Eq. (9).

***Application*** **3**. Consider an arbitrary *m* × *n* globe network of Fig. 1. When the current *J* flows into node *d*_1_(0, 0) and flows out node *d*_2_(*x*_2_, *m*), we have *y*_1_ = 0, *y*_2_ = *m*, Thus

41 $${S}_{{y}_{1},i}={S}_{0,i}=\,\sin ({y}_{1}{\theta }_{i})=0,\,{S}_{{y}_{2},i}=\,\sin (m{\theta }_{i})=0.$$

Substitutes Eq. (41) into Eq. (7), we get

42 $$\frac{{U}_{m\times n}(x,y)}{J}=-\,\frac{{r}_{0}}{n}y,\,(0\le y\le m)$$

When the current *J* flows past the network from node *d*_2_(*x*_2_, *m*) to node *d*_1_(0, 0), from Eq. (7), we obtain

43 $$\frac{{U}_{m\times n}(x,y)}{J}=\frac{{r}_{0}}{n}y,\,(0\le y\le m)$$

Eq. (42) and Eq. (43) tell us that the input and output place of current *J* affects the expression of the potential since we select *U*(0, 0) = 0.

***Application*** **4**. Consider an arbitrary *m* × *n* globe network of Fig. 1. When the current *J* goes from node *d*_1_(*x*_1_, *y*_1_) to node *d*_2_(*x*_2_, *y*_1_)(*y*_2_ = *y*_1_), from Eq. (7), the potential of an arbitrary node *d*(*x*, *y*) is

44 $$\frac{{U}_{m\times n}(x,y)}{J}=\frac{2r}{m}\sum _{i=2}^{m}(\frac{{\beta }_{{x}_{1},x}^{(i)}-{\beta }_{{x}_{2},x}^{(i)}}{{\lambda }_{i}^{n}+{\bar{\lambda }}_{i}^{n}-2}){S}_{{y}_{1},i}{S}_{y,i}.$$

***Application*** **5**. Consider an *m* × *n* globe network as shown in Fig. 1. Assuming the current injected to node *d*_*k*_(*x*_*k*_, *y*_1_) (*k* = 1, 2, … *k*) on the same latitude is *J*/*k*, the current outputted from the south pole *O*(0, 0) is *J*, by (39) we have the potential function

45 $$\frac{{U}_{m\times n}(x,y)}{J}={r}_{0}\frac{y{y}_{1}}{mn}+\frac{2r}{km}\sum _{i=2}^{m}\frac{{S}_{{y}_{1},i}{S}_{y,i}}{{\lambda }_{i}^{n}+{\bar{\lambda }}_{i}^{n}-2}\sum _{s=1}^{k}{\beta }_{{x}_{s},x}^{(i)}.$$

In particular, if the node *d*_*k*_(*x*_*k*_, *y*_1_)(*k* = 1, 2, … *n*) is evenly distributed on the same latitude line of the *n* nodes, then the potential at node *d*(*x*, *y*) is

46 $$\frac{{U}_{m\times n}(x,y)}{J}={r}_{0}\frac{y{y}_{1}}{mn}+\frac{{r}_{0}}{nm}\sum _{i=2}^{m}\frac{\sin ({y}_{1}{\theta }_{i})}{1-\,\cos \,{\theta }_{i}}\,\sin (y{\theta }_{i}).$$

where $\sum _{k=1}^{n}{\beta }_{{x}_{k},x}^{(i)}=\sum _{k=1}^{n}({F}_{n-|{x}_{k}-x|}^{(i)}+{F}_{|{x}_{k}-x|}^{(i)})=\frac{{\lambda }_{i}^{n}+{\bar{\lambda }}_{i}^{n}-2}{2b(1-\,\cos \,{\theta }_{i})}$ is used.

***Application*** **6**. Consider an arbitrary *m* × *n* globe network as shown in Fig. 1, we have the effective resistance between *d*_1_(*x*_1_, *y*_1_) and *d*_2_(*x*_2_, *y*_2_)

47 $${R}_{m\times n}({d}_{1},{d}_{2})=\frac{{({y}_{2}-{y}_{1})}^{2}}{mn}{r}_{0}+\frac{2r}{m}\sum _{i=2}^{m}\frac{{F}_{n}^{(i)}({S}_{1,i}^{2}+{S}_{2,i}^{2})-2({F}_{{\rm{\Delta }}x}^{(i)}+{F}_{n-{\rm{\Delta }}x}^{(i)}){S}_{1,i}{S}_{2,i}}{{\lambda }_{i}^{n}+{\bar{\lambda }}_{i}^{n}-2},$$

where Δ*x* = |*x*_2_ − *x*_1_|, and ${S}_{k,i}=\,\sin ({y}_{k}{\theta }_{i})$, *θ*_*i*_ = (*i*−1)*π*/*m* and ${F}_{k}^{(i)}=({\lambda }_{i}^{k}-{\bar{\lambda }}_{i}^{k})/({\lambda }_{i}-{\bar{\lambda }}_{i})$.

#### Proof

**of Eq. (47)**. Assuming the potentials at nodes of *d*_1_(*x*_1_, *y*_1_) and *d*_2_(*x*_2_, *y*_2_) are *U*_1_ and *U*_2_, using Ohm’s law yields

48 $${R}_{m\times n}({d}_{1},{d}_{2})=\frac{1}{J}({U}_{1}-{U}_{2}).$$

Taking (*x*, *y*) = (*x*_1_, *y*_1_) and (*x*, *y*) = (*x*_2_, *y*_2_) into Eq. (7), we get

49 $$\frac{U({x}_{1},{y}_{1})}{J}={r}_{0}\frac{{y}_{1}({y}_{1}-{y}_{2})}{mn}+\frac{2r}{m}\sum _{i=2}^{m}(\frac{{\beta }_{{x}_{1},{x}_{1}}^{(i)}{S}_{{y}_{1},i}-{\beta }_{{x}_{2},{x}_{1}}^{(i)}{S}_{{y}_{2},i}}{{\lambda }_{i}^{n}+{\bar{\lambda }}_{i}^{n}-2}){S}_{{y}_{1},i},$$

50 $$\frac{U({x}_{2},{y}_{2})}{J}={r}_{0}\frac{{y}_{2}({y}_{1}-{y}_{2})}{mn}+\frac{2r}{m}\sum _{i=2}^{m}(\frac{{\beta }_{{x}_{1},{x}_{2}}^{(i)}{S}_{{y}_{1},i}-{\beta }_{{x}_{2},{x}_{2}}^{(i)}{S}_{{y}_{2},i}}{{\lambda }_{i}^{n}+{\bar{\lambda }}_{i}^{n}-2}){S}_{{y}_{2},i}.$$

Putting Eqs (49) and (50) into Eq. (48) yields Eq. (47), where ${\beta }_{{x}_{2},{x}_{2}}^{(i)}={\beta }_{{x}_{1},{x}_{1}}^{(i)}={F}_{n}^{(i)}$ is used.

***Application*** **7**. We can use the hyperbolic functions to express Eq. (47) after replacing the variables, such as

51 $${R}_{m\times n}({d}_{1},{d}_{2})={r}_{0}\frac{{({y}_{2}-{y}_{1})}^{2}}{mn}+\frac{r}{m}\sum _{i=2}^{m}\frac{\cosh (n{L}_{i})({S}_{1,i}^{2}+{S}_{2,i}^{2})-2\,\cosh \,[(n-2{\rm{\Delta }}x){L}_{i}]{S}_{1,i}{S}_{2,i}}{\sinh (2{L}_{i})\sinh (n{L}_{i})},$$

where ${L}_{i}=\frac{1}{2}\,\mathrm{ln}\,{\lambda }_{i}$, and *λ*_*i*_ is defined in Eq. (10), and Δ*x* = |*x*_2_−*x*_1_|.

In addition, when *x*_1_ = *x*_2_, formula (51) reduces to

52 $${R}_{m\times n}({d}_{1},{d}_{2})={r}_{0}\frac{{({y}_{2}-{y}_{1})}^{2}}{mn}+\frac{r}{m}\sum _{i=2}^{m}\frac{\coth (n{L}_{i})}{\sinh (2{L}_{i})}{({S}_{1,i}-{S}_{2,i})}^{2}.$$

when *y*_2_ = *y*_1_, formula (51) reduces to

53 $${R}_{m\times n}({d}_{1},{d}_{2})=\frac{4r}{m}\sum _{i=2}^{m}\frac{\sinh ({\rm{\Delta }}x{L}_{i})\sinh (n-{\rm{\Delta }}x){L}_{i}}{\sinh (2{L}_{i})\sinh (n{L}_{i})}{S}_{1,i}^{2},$$

where $\cosh (n{L}_{i})-\,\cosh \,[(n-2{\rm{\Delta }}x){L}_{i}]=2\,\sinh ({\rm{\Delta }}x{L}_{i})\sinh (n-{\rm{\Delta }}x){L}_{i}$ is used.

Please note that ref.^27^ has studied the effective resistance of the *m* × *n* globe network, and the formula given in ref.^27^ is as Eq. (51). But here our approach is based on the potential function which is different from ref.^27^. Clearly using potential function to derive the equivalent resistance is more easy then the other method.

**Figure 3:**
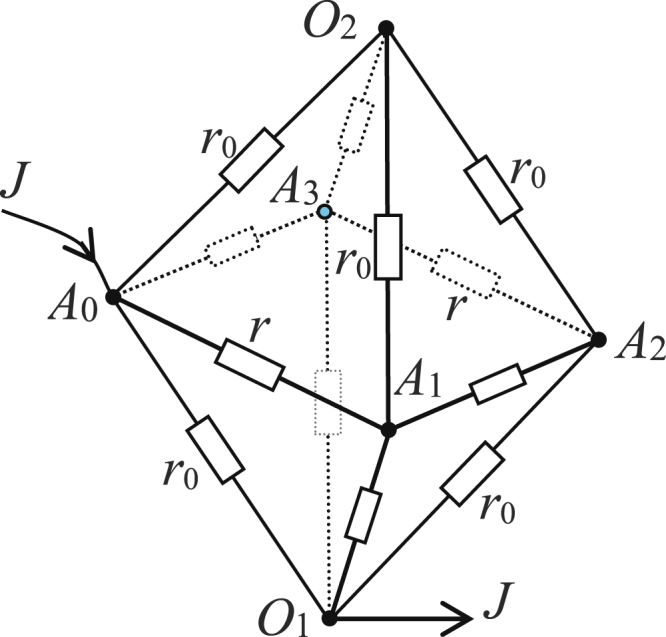
A simple 2× 4 globe lattice, where the unit resistor on each side of the quadrangle *A*_0_*A*_1_*A*_2_*A*_3_ is *r*, the other is *r*_0_.

***Application*** **8**. Consider a simple 2 × 4 lattice as shown in Fig. 3. Assuming the current *J* goes from *A*_0_ to *O*_1_, and selecting *U*(*O*_1_) = 0, by Eq. (7), we obtain the potentials of node *O*_2_ and *A*_*k*_ in the 2 × 4 resistor network,

54 $$U({O}_{2})=J\frac{1}{4}{r}_{0},$$

and

55 $$\frac{U({A}_{k})}{J}=\frac{1}{8}{r}_{0}+r\frac{{F}_{4-k}+{F}_{k}}{{\lambda }^{4}+{\bar{\lambda }}^{4}-2},$$

where $${F}_{k}=({\lambda }^{k}-{\bar{\lambda }}^{k})/(\lambda -\bar{\lambda })$$, *b* = *r*/*r*_0_, and $$\lambda =1+b+\sqrt{{b}^{2}+2b}$$, $$\bar{\lambda }=1+b-\sqrt{{b}^{2}+2b}$$.

From (55), we obtain three specific potential formulae

56 $$\frac{U({A}_{0})}{J}=\frac{1}{8}{r}_{0}+\frac{2{b}^{2}+4b+1}{4(1+b)({b}^{2}+2b)}r,$$

57 $$\frac{U({A}_{2})}{J}=\frac{1}{8}{r}_{0}+\frac{r}{4(1+b)({b}^{2}+2b)},$$

58 $$\frac{U({A}_{1})}{J}=\frac{U({A}_{3})}{J}=\frac{1}{8}{r}_{0}+\frac{r}{4({b}^{2}+2b)}.$$

In addition, by Eq. (47), we obtain effective resistance between nodes *A*_0_ and *O*_1_ and *A*_*k*_, *O*_1_ and *O*_2_ in the 2 × 4 resistor network,

59 $$R({A}_{k},{O}_{1})=\frac{1}{8}{r}_{0}+\frac{2{b}^{2}+4b+1}{4(1+b)({b}^{2}+2b)}r,$$

60 $$R({A}_{0},{A}_{1})=\frac{2{b}^{2}+3b}{2(1+b)({b}^{2}+2b)}r,$$

61 $$R({A}_{0},{A}_{2})=\frac{r}{1+b},$$

62 $$R({O}_{1},{O}_{2})=\frac{1}{2}{r}_{0},$$

By actual inspection and testing, the above Eqs (54–62) are in complete accord with the results of the actual circuit. Of course, the general potential formula (7) we achieved is inevitabl acoup sur since all of the computation and reduction are strict and self-consistent.

### A Summary

It is important for us to searching for the explicit potential formula of an arbitrary resistor network because resistor networks are often used to simulate the Laplace equation, but it has been a difficult thing to find the potential formula of the resistor network. This paper achieved a series of exact potential formulae in an *m* × *n* globe network by the *RT-I* method for the first time. Please note that RT method has two kinds of types which are respectively expressed by current parameters and potential parameters, such as in refs^36,37^ the RT method is expressed by potential parameters, but in our paper the RT method is expressed by current parameters.

As applications of the potential function in an *m* × *n* globe network, the resistance formula is derived naturally by potential function such as Eqs (47) and (51), and many interesting results are generated, such as Eqs (39 and 40), Eqs (42–46). Our results are helpful to resolve Laplace equation by modeling the resistor network, such as to resolve the potential function of static field and the temperature distribution of heat transfer function and so on.
